# *N*′-(3,4-Dimeth­oxy­benzyl­idene)-3,5-dihy­droxy­benzohydrazide methanol monosolvate

**DOI:** 10.1107/S1600536811029850

**Published:** 2011-07-30

**Authors:** Qi-Hui Zhang, Lian-Di Zhou, Chuan-Xun Li, Shan-Shan Huang, Bao-Jing Zhang

**Affiliations:** a College of Chemistry and Chemical Engineering, Chongqing University, Chongqing 400030, People’s Republic of China; b Department of Immunology, Basic Medical College, Chongqing Medical University, Chongqing 400016, People’s Republic of China; c School of Pharmacy, Dalian Medical University, Dalian 116044, People’s Republic of China

## Abstract

In the title compound, C_16_H_16_N_2_O_5_·CH_4_O, the two benzene rings in the Schiff base mol­ecule form a dihedral angle of 17.1 (1)°. In the crystal, inter­molecular O—H⋯O hydrogen bonds link the components into corrugated sheets parallel to the (101) plane.

## Related literature

For the crystal structures of related Schiff base compounds, see: Deng *et al.* (2009 ▶); Huang *et al.* (2008 ▶). For anti­bacterial and anti­tumor activities of Schiff base complexes, see: Brückner *et al.* (2000 ▶); Harrop *et al.* (2003 ▶); Ren *et al.* (2002 ▶).
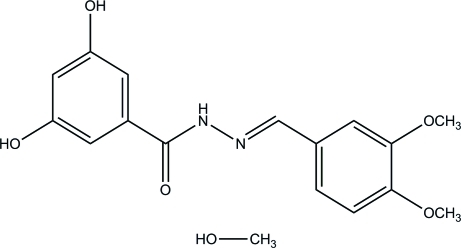


## Experimental

### 

#### Crystal data

C_16_H_16_N_2_O_5_·CH_4_O
*M* *_r_* = 348.35
Monoclinic, 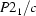

*a* = 12.467 (3) Å
*b* = 12.201 (3) Å
*c* = 11.149 (3) Å
β = 91.191 (3)°
*V* = 1695.5 (7) Å^3^
*Z* = 4
Mo *K*α radiation
μ = 0.10 mm^−1^
*T* = 295 K
0.22 × 0.18 × 0.15 mm

#### Data collection

Bruker APEXII CCD diffractometer
8351 measured reflections
2984 independent reflections
1794 reflections with *I* > 2σ(*I*)
*R*_int_ = 0.041

#### Refinement

*R*[*F* ^2^ > 2σ(*F* ^2^)] = 0.055
*wR*(*F* ^2^) = 0.162
*S* = 1.00
2984 reflections
236 parameters
H atoms treated by a mixture of independent and constrained refinement
Δρ_max_ = 0.68 e Å^−3^
Δρ_min_ = −0.32 e Å^−3^

### 

Data collection: *APEX2* (Bruker, 2007 ▶); cell refinement: *APEX2*; data reduction: *SAINT-Plus* (Bruker, 2007 ▶); program(s) used to solve structure: *SHELXTL* (Sheldrick, 2008 ▶); program(s) used to refine structure: *SHELXTL*; molecular graphics: *SHELXTL*; software used to prepare material for publication: *SHELXTL*.

## Supplementary Material

Crystal structure: contains datablock(s) I, global. DOI: 10.1107/S1600536811029850/cv5134sup1.cif

Structure factors: contains datablock(s) I. DOI: 10.1107/S1600536811029850/cv5134Isup2.hkl

Supplementary material file. DOI: 10.1107/S1600536811029850/cv5134Isup3.cml

Additional supplementary materials:  crystallographic information; 3D view; checkCIF report

## Figures and Tables

**Table 1:** Hydrogen-bond geometry (Å, °)

*D*—H⋯*A*	*D*—H	H⋯*A*	*D*⋯*A*	*D*—H⋯*A*
O2—H2⋯O6	0.82	1.92	2.728 (3)	168
O1—H1⋯O5^i^	0.82	1.93	2.742 (3)	172
O6—H6⋯O4^ii^	0.82	2.19	2.973 (4)	161

Symmetry codes: (i) 
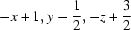
; (ii) 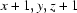.

**Fig. 1.**
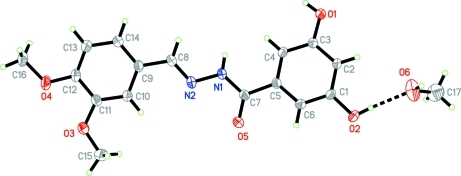
The molecular structure of (I) showing the atomic numbering and 30% probability displacement ellipsoids. Dashed line denotes hydrogen bond.

## supplementary crystallographic information

### Comment

Schiff bases containing the C=N fragments arouses considerable interest due to their antifungal, anticancer and antibacterial activities (Brückner *et al.*, 2000; Harrop *et al.*, 2003; Ren *et al.*, 2002). In continuation of our structural studies of Shiff base compounds (Deng *et al.*, 2009; Huang *et al.*, 2008) we present here the crystal structure of the title compound, (I).

In (I) (Fig. 1), the C7—O5 and C8—N2 bond lengths are 1.228 (3) and  1.274 (3) Å, respecitvely, corresponding to those reported for the related compounds (Deng *et al.*, 2009; Huang *et al.*, 2008). The solvent molecule is linked to the Shiff base molecule *via*  O—H···O hydrogen bond (Table 1).

In the crystal structure, intermolecular O—H···O hydrogen bonds (Table 1) link all moieties into corrugated sheets parallel to (101) plane.

### Experimental

3,5-Dihydroxybenzhydrazide (0.1 mmol,16.8 mg) and 3,4-dimethoxybenzaldehyde (0.1 mmol, 16.6 mg) were dissolved in a methanol solution (10 ml). The mixture was stirred at room temperature for 20 minutes and filtered. After keeping the filtrate in air for three days, colourless block-like crystals were formed.

### Refinement

The H18 atom bonded to N1 was located in a difference map and refined isotropically, Other H atoms were placed in geometrically idealized positions (C—H 0.93-0.97 Å; O—H 0.82 Å), and allowed to ride on their parent atoms, with *U*_iso_(H)=1.2-1.5 *U*_eq_ of the parent atom.

### Crystal data

**Table:**

C_16_H_16_N_2_O_5_·CH_4_O	*F*(000) = 736
*M**_r_* = 348.35	*D*_x_ = 1.365 Mg m^−^^3^
Monoclinic, *P*2_1_/*c*	Mo *K*α radiation, λ = 0.71073 Å
Hall symbol: -P2ybc	Cell parameters from 1340 reflections
*a* = 12.467 (3) Å	θ = 2.3–21.1°
*b* = 12.201 (3) Å	µ = 0.10 mm^−^^1^
*c* = 11.149 (3) Å	*T* = 295 K
β = 91.191 (3)°	Block, colourless
*V* = 1695.5 (7) Å^3^	0.22 × 0.18 × 0.15 mm
*Z* = 4	

### Data collection

**Table:**

Bruker APEXII CCD diffractometer	1794 reflections with *I* > 2σ(*I*)
Radiation source: fine-focus sealed tube	*R*_int_ = 0.041
graphite	θ_max_ = 25.0°, θ_min_ = 2.3°
phi and ω scans	*h* = −14→11
8351 measured reflections	*k* = −14→12
2984 independent reflections	*l* = −12→13

### Refinement

**Table:**

Refinement on *F*^2^	Primary atom site location: structure-invariant direct methods
Least-squares matrix: full	Secondary atom site location: difference Fourier map
*R*[*F*^2^ > 2σ(*F*^2^)] = 0.055	Hydrogen site location: inferred from neighbouring sites
*wR*(*F*^2^) = 0.162	H atoms treated by a mixture of independent and constrained refinement
*S* = 1.00	*w* = 1/[σ^2^(*F*_o_^2^) + (0.0897*P*)^2^] where *P* = (*F*_o_^2^ + 2*F*_c_^2^)/3
2984 reflections	(Δ/σ)_max_ < 0.001
236 parameters	Δρ_max_ = 0.68 e Å^−^^3^
0 restraints	Δρ_min_ = −0.32 e Å^−^^3^

### Special details

**Table:**

Geometry. All e.s.d.'s (except the e.s.d. in the dihedral angle between two l.s. planes) are estimated using the full covariance matrix. The cell e.s.d.'s are taken into account individually in the estimation of e.s.d.'s in distances, angles and torsion angles; correlations between e.s.d.'s in cell parameters are only used when they are defined by crystal symmetry. An approximate (isotropic) treatment of cell e.s.d.'s is used for estimating e.s.d.'s involving l.s. planes.
Refinement. Refinement of *F*^2^ against ALL reflections. The weighted *R*-factor *wR* and goodness of fit *S* are based on *F*^2^, conventional *R*-factors *R* are based on *F*, with *F* set to zero for negative *F*^2^. The threshold expression of *F*^2^ > σ(*F*^2^) is used only for calculating *R*-factors(gt) *etc*. and is not relevant to the choice of reflections for refinement. *R*-factors based on *F*^2^ are statistically about twice as large as those based on *F*, and *R*- factors based on ALL data will be even larger.

### Fractional atomic coordinates and isotropic or equivalent isotropic displacement parameters (Å^2^)

**Table:**

	*x*	*y*	*z*	*U*_iso_*/*U*_eq_	
H18	0.492 (3)	0.340 (3)	0.619 (3)	0.067 (11)*	
N2	0.38492 (18)	0.42464 (18)	0.54230 (19)	0.0425 (6)	
O5	0.44125 (17)	0.56294 (14)	0.72026 (18)	0.0515 (5)	
O1	0.65292 (18)	0.18020 (14)	0.96344 (19)	0.0601 (7)	
H1	0.6297	0.1410	0.9091	0.090*	
O2	0.70583 (18)	0.55400 (15)	1.06156 (18)	0.0544 (6)	
H2	0.7458	0.5258	1.1121	0.082*	
N1	0.4573 (2)	0.3984 (2)	0.6318 (2)	0.0430 (6)	
C8	0.3653 (2)	0.3502 (2)	0.4648 (2)	0.0421 (7)	
H8	0.4011	0.2835	0.4716	0.051*	
C9	0.2892 (2)	0.3658 (2)	0.3669 (2)	0.0402 (7)	
C4	0.5705 (2)	0.3194 (2)	0.8382 (2)	0.0390 (7)	
H4	0.5406	0.2673	0.7867	0.047*	
C6	0.5969 (2)	0.5075 (2)	0.8954 (2)	0.0387 (7)	
H6A	0.5834	0.5817	0.8830	0.046*	
C7	0.4798 (2)	0.4703 (2)	0.7203 (2)	0.0373 (6)	
O3	0.10563 (17)	0.57181 (16)	0.23529 (19)	0.0611 (6)	
O4	0.06565 (17)	0.41280 (18)	0.09236 (18)	0.0589 (6)	
C1	0.6621 (2)	0.4745 (2)	0.9900 (2)	0.0381 (7)	
C2	0.6813 (2)	0.3649 (2)	1.0103 (2)	0.0411 (7)	
H2A	0.7253	0.3430	1.0743	0.049*	
C5	0.5515 (2)	0.43046 (19)	0.8190 (2)	0.0348 (6)	
C14	0.2689 (2)	0.2816 (2)	0.2869 (2)	0.0450 (7)	
H14	0.3055	0.2156	0.2958	0.054*	
C11	0.1620 (2)	0.4774 (2)	0.2587 (2)	0.0423 (7)	
C10	0.2352 (2)	0.4653 (2)	0.3514 (2)	0.0411 (7)	
H10	0.2488	0.5232	0.4038	0.049*	
C12	0.1404 (2)	0.3909 (2)	0.1795 (2)	0.0443 (7)	
C3	0.6346 (2)	0.2874 (2)	0.9350 (2)	0.0400 (7)	
C13	0.1950 (2)	0.2940 (2)	0.1939 (3)	0.0479 (8)	
H13	0.1821	0.2365	0.1409	0.058*	
C16	0.0472 (3)	0.3318 (3)	0.0018 (3)	0.0626 (9)	
H16A	0.1120	0.3200	−0.0414	0.094*	
H16B	−0.0083	0.3565	−0.0527	0.094*	
H16C	0.0255	0.2644	0.0386	0.094*	
C15	0.1393 (3)	0.6683 (3)	0.2954 (3)	0.0755 (11)	
H15A	0.1316	0.6589	0.3803	0.113*	
H15B	0.0961	0.7289	0.2684	0.113*	
H15C	0.2132	0.6825	0.2783	0.113*	
C17	0.8698 (4)	0.4724 (4)	1.3338 (3)	0.0897 (13)	
H17A	0.9159	0.5304	1.3616	0.135*	
H17B	0.8985	0.4032	1.3598	0.135*	
H17C	0.7995	0.4821	1.3658	0.135*	
O6	0.8632 (2)	0.4745 (3)	1.2114 (3)	0.1058 (10)	
H6	0.9237	0.4726	1.1841	0.159*	

### Atomic displacement parameters (Å^2^)

**Table:**

	*U*^11^	*U*^22^	*U*^33^	*U*^12^	*U*^13^	*U*^23^
N2	0.0467 (14)	0.0457 (14)	0.0346 (13)	0.0035 (11)	−0.0118 (11)	0.0033 (11)
O5	0.0674 (14)	0.0377 (11)	0.0485 (12)	0.0063 (10)	−0.0173 (10)	0.0032 (9)
O1	0.0853 (17)	0.0318 (11)	0.0617 (14)	0.0043 (10)	−0.0389 (12)	−0.0004 (9)
O2	0.0710 (15)	0.0387 (11)	0.0526 (13)	−0.0065 (10)	−0.0229 (11)	−0.0088 (9)
N1	0.0514 (15)	0.0414 (14)	0.0354 (13)	0.0094 (12)	−0.0155 (11)	−0.0022 (11)
C8	0.0459 (17)	0.0409 (15)	0.0392 (16)	0.0054 (13)	−0.0074 (13)	0.0040 (13)
C9	0.0420 (17)	0.0450 (16)	0.0333 (15)	−0.0011 (13)	−0.0075 (13)	0.0039 (12)
C4	0.0471 (17)	0.0339 (14)	0.0356 (15)	−0.0042 (12)	−0.0097 (13)	−0.0023 (11)
C6	0.0468 (17)	0.0297 (14)	0.0394 (16)	−0.0025 (12)	−0.0043 (13)	0.0010 (12)
C7	0.0410 (16)	0.0354 (15)	0.0355 (15)	0.0004 (12)	−0.0028 (13)	0.0057 (12)
O3	0.0666 (15)	0.0524 (13)	0.0629 (14)	0.0191 (11)	−0.0278 (11)	−0.0073 (10)
O4	0.0559 (13)	0.0718 (14)	0.0479 (12)	0.0096 (11)	−0.0243 (10)	−0.0095 (10)
C1	0.0433 (16)	0.0349 (15)	0.0360 (15)	−0.0068 (12)	−0.0062 (13)	−0.0050 (12)
C2	0.0478 (17)	0.0369 (15)	0.0380 (15)	0.0007 (13)	−0.0156 (13)	−0.0012 (12)
C5	0.0363 (15)	0.0355 (14)	0.0324 (14)	−0.0020 (12)	−0.0049 (12)	0.0033 (11)
C14	0.0537 (18)	0.0400 (15)	0.0409 (16)	0.0041 (14)	−0.0065 (14)	−0.0002 (13)
C11	0.0410 (16)	0.0454 (16)	0.0402 (16)	0.0069 (13)	−0.0064 (13)	0.0011 (13)
C10	0.0430 (16)	0.0439 (16)	0.0361 (15)	0.0023 (13)	−0.0068 (13)	−0.0018 (12)
C12	0.0403 (17)	0.0546 (18)	0.0376 (16)	−0.0019 (14)	−0.0088 (13)	0.0007 (13)
C3	0.0464 (17)	0.0322 (15)	0.0407 (16)	−0.0013 (12)	−0.0113 (13)	0.0009 (12)
C13	0.0561 (19)	0.0460 (17)	0.0412 (17)	−0.0029 (14)	−0.0088 (15)	−0.0047 (13)
C16	0.069 (2)	0.071 (2)	0.0461 (18)	−0.0068 (18)	−0.0232 (17)	−0.0041 (16)
C15	0.091 (3)	0.051 (2)	0.083 (3)	0.0223 (19)	−0.022 (2)	−0.0127 (19)
C17	0.108 (4)	0.100 (3)	0.061 (3)	−0.003 (3)	0.011 (2)	−0.002 (2)
O6	0.077 (2)	0.151 (3)	0.088 (2)	0.007 (2)	−0.0210 (16)	0.011 (2)

### Geometric parameters (Å, °)

**Table:**

N2—C8	1.274 (3)	O4—C16	1.429 (3)
N2—N1	1.370 (3)	C1—C2	1.376 (4)
O5—C7	1.228 (3)	C2—C3	1.384 (4)
O1—C3	1.364 (3)	C2—H2A	0.9300
O1—H1	0.8200	C14—C13	1.381 (4)
O2—C1	1.362 (3)	C14—H14	0.9300
O2—H2	0.8200	C11—C10	1.372 (4)
N1—C7	1.345 (3)	C11—C12	1.399 (4)
N1—H18	0.85 (3)	C10—H10	0.9300
C8—C9	1.443 (4)	C12—C13	1.371 (4)
C8—H8	0.9300	C13—H13	0.9300
C9—C14	1.380 (4)	C16—H16A	0.9600
C9—C10	1.398 (4)	C16—H16B	0.9600
C4—C3	1.385 (4)	C16—H16C	0.9600
C4—C5	1.391 (3)	C15—H15A	0.9600
C4—H4	0.9300	C15—H15B	0.9600
C6—C1	1.379 (4)	C15—H15C	0.9600
C6—C5	1.382 (3)	C17—O6	1.366 (4)
C6—H6A	0.9300	C17—H17A	0.9600
C7—C5	1.486 (4)	C17—H17B	0.9600
O3—C11	1.371 (3)	C17—H17C	0.9600
O3—C15	1.414 (4)	O6—H6	0.8200
O4—C12	1.360 (3)		
			
C8—N2—N1	116.2 (2)	O3—C11—C10	124.3 (2)
C3—O1—H1	109.5	O3—C11—C12	115.0 (2)
C1—O2—H2	109.5	C10—C11—C12	120.7 (2)
C7—N1—N2	120.4 (2)	C11—C10—C9	119.9 (2)
C7—N1—H18	125 (2)	C11—C10—H10	120.1
N2—N1—H18	114 (2)	C9—C10—H10	120.1
N2—C8—C9	122.2 (3)	O4—C12—C13	125.8 (3)
N2—C8—H8	118.9	O4—C12—C11	115.0 (2)
C9—C8—H8	118.9	C13—C12—C11	119.2 (3)
C14—C9—C10	119.0 (2)	O1—C3—C2	116.6 (2)
C14—C9—C8	120.0 (2)	O1—C3—C4	122.8 (2)
C10—C9—C8	121.0 (2)	C2—C3—C4	120.6 (2)
C3—C4—C5	119.2 (2)	C12—C13—C14	120.3 (3)
C3—C4—H4	120.4	C12—C13—H13	119.9
C5—C4—H4	120.4	C14—C13—H13	119.9
C1—C6—C5	120.0 (2)	O4—C16—H16A	109.5
C1—C6—H6A	120.0	O4—C16—H16B	109.5
C5—C6—H6A	120.0	H16A—C16—H16B	109.5
O5—C7—N1	121.6 (2)	O4—C16—H16C	109.5
O5—C7—C5	122.1 (2)	H16A—C16—H16C	109.5
N1—C7—C5	116.3 (2)	H16B—C16—H16C	109.5
C11—O3—C15	117.6 (2)	O3—C15—H15A	109.5
C12—O4—C16	117.8 (2)	O3—C15—H15B	109.5
O2—C1—C2	122.0 (2)	H15A—C15—H15B	109.5
O2—C1—C6	117.5 (2)	O3—C15—H15C	109.5
C2—C1—C6	120.5 (2)	H15A—C15—H15C	109.5
C1—C2—C3	119.6 (2)	H15B—C15—H15C	109.5
C1—C2—H2A	120.2	O6—C17—H17A	109.5
C3—C2—H2A	120.2	O6—C17—H17B	109.5
C6—C5—C4	120.1 (2)	H17A—C17—H17B	109.5
C6—C5—C7	117.8 (2)	O6—C17—H17C	109.5
C4—C5—C7	122.1 (2)	H17A—C17—H17C	109.5
C9—C14—C13	120.9 (3)	H17B—C17—H17C	109.5
C9—C14—H14	119.5	C17—O6—H6	109.5
C13—C14—H14	119.5		
			
C8—N2—N1—C7	−177.3 (2)	C15—O3—C11—C10	12.5 (4)
N1—N2—C8—C9	178.8 (2)	C15—O3—C11—C12	−167.1 (3)
N2—C8—C9—C14	−178.5 (3)	O3—C11—C10—C9	−179.4 (3)
N2—C8—C9—C10	1.4 (4)	C12—C11—C10—C9	0.2 (4)
N2—N1—C7—O5	−4.0 (4)	C14—C9—C10—C11	1.0 (4)
N2—N1—C7—C5	174.5 (2)	C8—C9—C10—C11	−178.9 (3)
C5—C6—C1—O2	179.2 (2)	C16—O4—C12—C13	−5.1 (4)
C5—C6—C1—C2	−1.0 (4)	C16—O4—C12—C11	174.2 (3)
O2—C1—C2—C3	179.8 (3)	O3—C11—C12—O4	−1.0 (4)
C6—C1—C2—C3	0.1 (4)	C10—C11—C12—O4	179.4 (3)
C1—C6—C5—C4	0.8 (4)	O3—C11—C12—C13	178.3 (3)
C1—C6—C5—C7	178.6 (2)	C10—C11—C12—C13	−1.3 (4)
C3—C4—C5—C6	0.4 (4)	C1—C2—C3—O1	−177.1 (3)
C3—C4—C5—C7	−177.2 (2)	C1—C2—C3—C4	1.2 (4)
O5—C7—C5—C6	−16.5 (4)	C5—C4—C3—O1	176.7 (3)
N1—C7—C5—C6	164.9 (2)	C5—C4—C3—C2	−1.4 (4)
O5—C7—C5—C4	161.2 (3)	O4—C12—C13—C14	−179.6 (3)
N1—C7—C5—C4	−17.4 (4)	C11—C12—C13—C14	1.2 (4)
C10—C9—C14—C13	−1.1 (4)	C9—C14—C13—C12	0.1 (4)
C8—C9—C14—C13	178.7 (3)		

### Hydrogen-bond geometry (Å, °)

**Table:**

*D*—H···*A*	*D*—H	H···*A*	*D*···*A*	*D*—H···*A*
O2—H2···O6	0.82	1.92	2.728 (3)	168.
O1—H1···O5^i^	0.82	1.93	2.742 (3)	172.
O6—H6···O4^ii^	0.82	2.19	2.973 (4)	161.

Symmetry codes: (i) −*x*+1, *y*−1/2, −*z*+3/2; (ii) *x*+1, *y*, *z*+1.
